# Supplementary material for: Cumulative semantic interference is blind to morphological complexity and originates at the conceptual level
Source: PLoS One. 2022 Jun 9;17(6):e0268915. doi: 10.1371/journal.pone.0268915 (PMC9182628; doi:10.1371/journal.pone.0268915)
Supplement: S1 Appendix — (DOCX) [file pone.0268915.s001.docx]

**S1 Appendix: Material**

| **Category** | **Item No** | |  |  |  |  |
| --- | --- | --- | --- | --- | --- | --- |
|  | **1** | | **2** | **3** | **4** | **5** |
| **Accessoires**  **(accessories)** | | Halstuch  *neck cloth*  (neckerchief) | Strohhut  (straw hat) | Schirmmütze  (visor cap) | Sonnenbrille  (sunglasses) | Wollschal  (wool scarf) |
| **Bürobedarf**  **(stationary)** | | Büroklammer  *office clip*  (paperclip) | Notizzettel  *note paper*  (notepad) | Filzstift  (felt pen) | Holzlineal  *wood ruler*  (wooden ruler) | Papierschere  (paper scissors) |
| **Gebäude**  **(buildings)** | | Gartenlaube  (garden shed) | Skihütte  (ski hut) | Windmühle  (windmill) | Ritterburg  (knight’s castle) | Stadtvilla  (urban villa) |
| **Gemüse**  **(vegetables)** | | Kopfsalat  *head lettuce*  (lettuce head) | Staudensellerie  (perennial celery) | Fleischtomate  *flesh tomato*  (beef tomato*)* | Chinakohl  (napa cabbage) | Frühlingszwiebel  (spring onion) |
| **Geschirr**  **(crockery)** | | Suppenteller  (soup plate) | Teetasse  (tea cup) | Sektglas  (champagne glass) | Kaffeebecher  (coffee mug) | Müslischüssel  (muesli bowl) |
| **Getränke**  **(drinks)** | | Orangensaft  (orange juice) | Malzbier  (malt beer) | Apfelschorle  (apple spritzer) | Mineralwasser  (mineral water) | Dessertwein  (dessert wine) |
| **Insekten**  **(insects)** | | Kellerassel  (pill bug) | Maikäfer  (maybug) | Honigbiene  (honey bee) | Blattlaus  *leaf louse*  (plant louse) | Kreuzspinne  (cross spider) |
| **Kleidung**  **(apparel)** | | Lederhose  (leather pants) | Wintermantel  (winter coat) | Seidenbluse  (satin blouse) | Herrenhemd  (man’s shirt) | Faltenrock  (plaid skirt) |
| **Körperteile**  **(body parts)** | | Milchzahn  (milk tooth) | Hakennase  (hooknose) | Mittelfinger  (middle finger) | Kussmund  *kiss mouth*  (puckered lips) | Babybauch  (baby belly) |
| **Lebensmittel**  **(food)** | | Schimmelkäse  (mould cheese) | Kräuterquark  (herb quark) | Blutwurst  (blood sausage) | Parmaschinken  (parma ham) | Rindersalami  (beef salami) |
| **Meerestiere**  **(marine animals)** | | Königkrabbe  (king crab) | Kugelfisch  (blowfish) | Hammerhai  (hammerhead shark) | Buckelwal  (hunchback whale) | Feuerqualle  (hair jelly) |
| **Möbel**  **(furniture)** | | Küchentisch  (kitchen table) | Bücherregal  (book shelf) | Kleiderschrank  *clothes cupboard*  (wardrobe) | Ohrensessel  (wing chair) | Himmelbett  (canopy bed) |
| **Pflanzen**  **(plants)** | | Birnbaum  (pear tree) | Fliegenpilz  *fly mushroom*  (fly agaric) | Glockenblume  (bellflower) | Kokospalme  (coco palm) | Dornenbusch  (pricky shrub) |
| **Säugetiere**  **(mammals)** | | Zwergmaus  (pygmy mouse) | Hauskatze  (pet cat) | Schäferhund  (sheperd dog) | Feldhase  (brown hare) | Goldhamster  (golden hamster) |
| **Süßspeisen**  **(confectionary)** | | Streuselkuchen  (crumble cake) | Sahnetorte  (cream tart) | Vanillepudding  (vanilla pudding) | Butterkeks  (butter biscuit) | Zitroneneis  (lemon icecream) |
| **Trage- behälter**  **(bags)** | | Handtasche  (handbag) | Schulranzen  (school bag) | Jutebeutel  (jute bag) | Reisekoffer  (suitcase) | Plastiktüte  (plastic bag) |
| **Transport- mittel**  **(transportation)** | | Schlauchboot  (rubber dinghy) | Segelschiff  (sail boat) | Pferdekutsche  (horse carriage) | Kastenwagen  (panel truck) | Düsenjet  (jet plane) |
| **Werkzeuge**  **(tools)** | | Kreissäge  (chain saw) | Gummihammer  (rubber mallet) | Eisennagel  (iron nail) | Rohrzange  (pipe wrench) | Metalldübel  (metall dowel) |
| **Filler** | | Presentationflöte (recorder), Zahlenschloss (combination lock), Kassenbon (receipt)  Laserdrucker (laser printer), Türschlüssel (door key), Mülltonne (rubbish bin), Pappkarton (paper carton), Landkarte (map), Bassgitarre (bass guitar), Silbermünze (silver coin), Schiefertafel (slate), Federkissen (feather pillow), Haarbürste (hairbrush), Geldschein (bankote), Radiowecker (clock radio), Pulverschnee (powdersnow), Felsküste (rocky shore), Kieselstein (pebble), Wirbelsturm (tornado), Sandwüste (sand desert), Storchennest (stork’s nest), Bodennebel (mist), Sichelmond (crescent moon), Kugelblitz (ball lightning), Heuballen (bale of hay), Sommerwiese (meadow), Komposterde (compost soil), Regenpfütze (rain puddle), Bergsee (mountain lake), Vulkaninsel (volcanic island) | | | | |
