# Supplementary material for: Cumulative semantic interference is blind to morphological complexity and originates at the conceptual level
Source: PLoS One. 2022 Jun 9;17(6):e0268915. doi: 10.1371/journal.pone.0268915 (PMC9182628; doi:10.1371/journal.pone.0268915)
Supplement: S2 Appendix — (DOCX) [file pone.0268915.s002.docx]

**S2 Appendix**

| **S1 Table. Predicting cumulative facilitation with cumulative interference.** Model: log(Facilitation effect) ~ Interference effect*Presentation+Interference effect*Semantic similarity + Interference effect: Presentation + Interference effect *Word type +(1\|Subject) | | | | |
| --- | --- | --- | --- | --- |
|  | **log(ec)** | | | |
| *Predictors* | *Estimates* | *std. Error* | *t-value* | *p* |
| (Intercept) | 7.589 | 0.003 | 2961.505 | **<0.001** |
| Interference effect | 0.000 | 0.000 | 2.357 | **0.019** |
| Presentation | 0.004 | 0.001 | 2.940 | **0.003** |
| Semantic similarity | 0.005 | 0.004 | 1.141 | 0.254 |
| Word type | 0.006 | 0.004 | 1.457 | 0.145 |
| Interference effect : Presentation | -0.000 | 0.000 | -0.693 | 0.489 |
| Interference effect: Semantic similarity | -0.000 | 0.000 | -2.081 | **0.038** |
| Interference effect: Word type | -0.000 | 0.000 | -2.152 | **0.032** |
| Interference effect: Presentation : Semantic similarity | 0.000 | 0.000 | 3.953 | **<0.001** |
| **Random Effects** | | | | |
| σ^2^ | 0.01 | | | |
| τ_00_ _subject_ | 0.00 | | | |
| ICC _subject_ | 0.01 | | | |
| Observations | 2375 | | | |
| Marginal R^2^ / Conditional R^2^ | 0.017 / 0.031 | | | |

| \| **S2 Table. Model output for naming task after the classification task**. Model: 1000/RT ~ Word type*Ordinal position+ Ordinal position *Semantic similarity + (Word type \|\|Subject) + (1\| Category)+( Word type \|\| Item) \| \| \| \| \| \| --- \| --- \| --- \| --- \| --- \| \|  \| **1000/RT** \| \| \| \| \| *Predictors* \| *Estimates* \| *std. Error* \| *t-value* \| *p* \| \| (Intercept) \| 1.4693 \| 0.0337 \| 43.5484 \| **<0.001** \| \| Word type \| -0.2371 \| 0.0236 \| -10.0553 \| **<0.001** \| \| Ordinal position \| 0.0568 \| 0.0154 \| 3.6750 \| **<0.001** \| \| Semantic similarity \| -0.0264 \| 0.0269 \| -0.9833 \| 0.328 \| \| Word type * Ordinal position \| 0.0113 \| 0.0227 \| 0.4965 \| 0.620 \| \| Ordinal position * Semantic similarity \| -0.0336 \| 0.0220 \| -1.5313 \| 0.126 \| \| **Random Effects** \| \| \| \| \| \| σ^2^ \| 0.06 \| \| \| \| \| τ_00_ _item_ \| 0.01 \| \| \| \| \| τ_00_ _item_ \| 0.02 \| \| \| \| \| τ_00_ _subject_ \| 0.01 \| \| \| \| \| τ_00_ _subject.1_ \| 0.03 \| \| \| \| \| τ_00_ _category_ \| 0.00 \| \| \| \| \| ICC _item_ \| 0.11 \| \| \| \| \| ICC _item_ \| 0.12 \| \| \| \| \| ICC _subject_ \| 0.06 \| \| \| \| \| ICC _subject.1_ \| 0.21 \| \| \| \| \| ICC _category_ \| 0.01 \| \| \| \| |
| --- | --- | --- | --- | --- | --- | --- | --- | --- | --- | --- | --- | --- | --- | --- | --- | --- | --- | --- | --- | --- | --- | --- | --- | --- | --- | --- | --- | --- | --- | --- | --- | --- | --- | --- | --- | --- | --- | --- | --- | --- | --- | --- | --- | --- | --- | --- | --- | --- | --- | --- | --- | --- | --- | --- | --- | --- | --- | --- | --- | --- | --- | --- | --- | --- | --- | --- | --- | --- | --- | --- | --- | --- | --- | --- | --- | --- | --- | --- | --- | --- | --- | --- | --- | --- | --- | --- | --- | --- | --- | --- | --- | --- | --- | --- | --- | --- | --- | --- | --- | --- | --- | --- | --- | --- | --- |


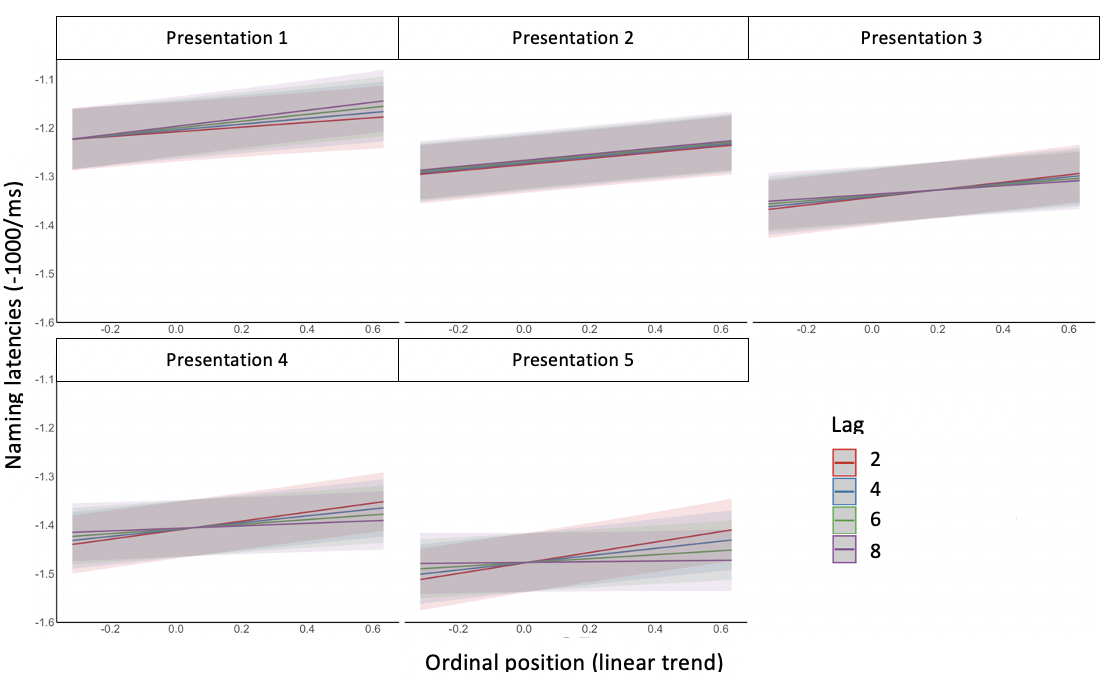


**S1 Fig. Predicted influence of Lag on the Interference effect (Ordinal position) observed in Exp1 broken down by presentation.** Short lags induce greater interference than longer ones only in Presentation 5.


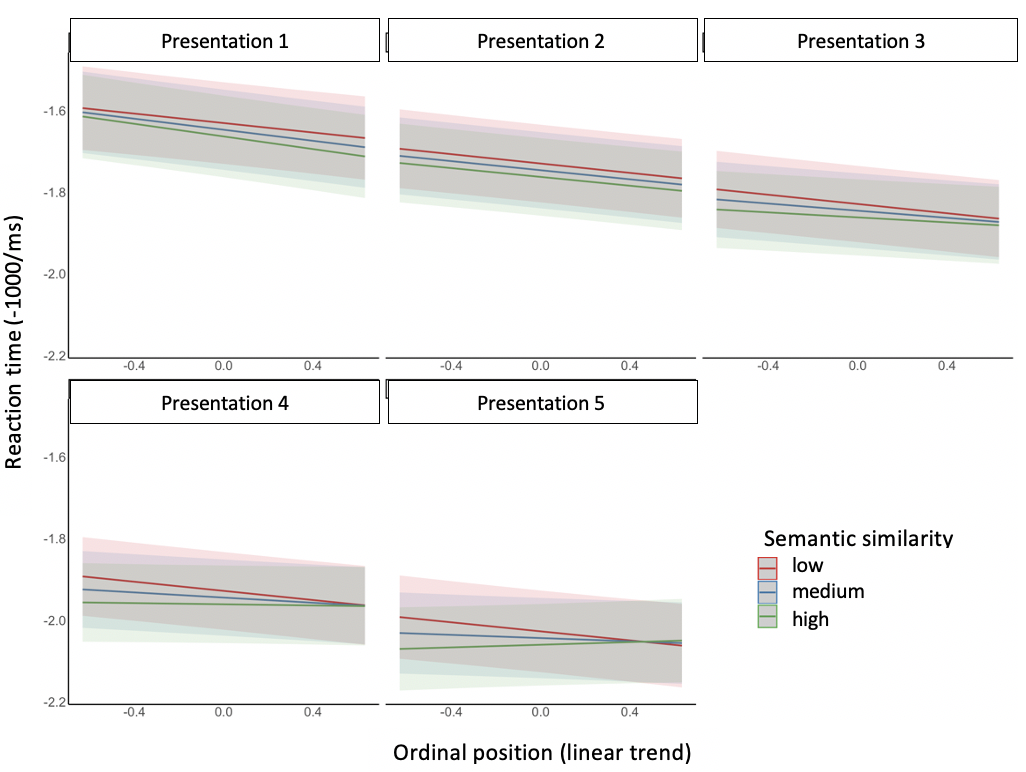


**S2 Fig. Predicted influence of semantic similarity on cumulative facilitation (inverse transformation), broken**

**down by presentation**.


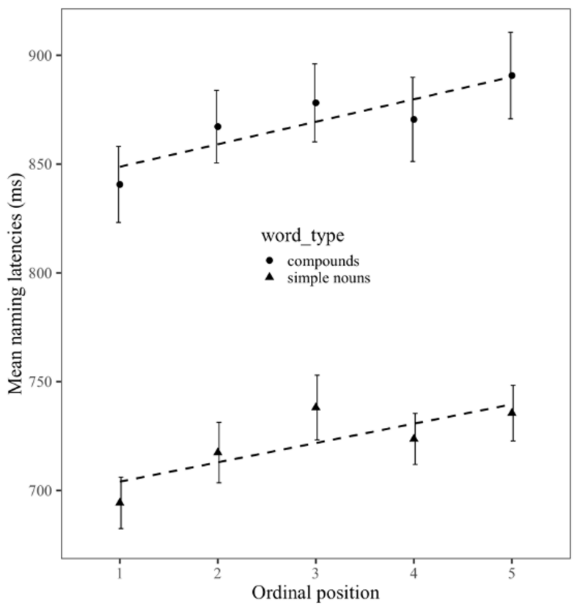


**S3 Fig. Mean reaction times (naming latency) and standard error (in milliseconds) observed in the check task (single naming cycle after Experiment 2) broken down by Ordinal position and Word type.**
