# Supplementary material for: Cumulative semantic interference is blind to morphological complexity and originates at the conceptual level
Source: PLoS One. 2022 Jun 9;17(6):e0268915. doi: 10.1371/journal.pone.0268915 (PMC9182628; doi:10.1371/journal.pone.0268915)
Supplement: Attachment — Submitted filename: Response_to_Reviewers.docx [file pone.0268915.s003.docx]

Reviewer #1:

In this paper, Döring and colleagues report a study investigating whether morphologically complex noun-noun compounds elicit cumulative semantic interference (CSI) in continuous naming and cumulative semantic facilitation/priming in man-made/natural decisions similarly to simple nouns. In both experiments, results were similar to those observed for simple nouns. As the authors themselves note, given that previous studies with simple nouns (e.g., Belke, 2013; Riley et al., 2014) have shown the origin of the CSI effect is at the conceptual level and its locus at the lexical level, one could argue that morphology is largely irrelevant to the argument concerning its origin but potentially relevant to its lexical locus. However, I’m not convinced the experiments do provide a straightforward test of whether activation patterns of compounds differ from simple nouns, particularly as some discrepant results need further explanation.

| **Reviewer’s comments** | **Authors’ responses** |
| --- | --- |
| Major concerns  The motivation for conducting the study emphasises different patterns of CSI might arise due to the single vs. multiple lemma account of compounds. The authors’ preferred hypothesis is that compounds will elicit weaker CSI as constituent lemmas become activated, including those of the unrelated modifiers that will essentially add noise to the system. The authors seem to have previously addressed the multiple lemma issue in their recent publication that they cite but don’t discuss in detail when motivating the present study (see doi link below). That study showed the constituent lemmas of compounds are activated and that significantly weaker CSI occurs for compounds than simple nouns – as predicted in the present study. So, is the finding of no difference in magnitude of CSI between compounds and simple nouns in Experiment 1 then a failed replication of this previous result? If so, this should be addressed squarely in both the Introduction and Discussion.  Döring, A.-L., Abdel Rahman, R., Zwitserlood, P., & Lorenz, A. (2021). On the lexical representation(s) of compounds: A continuous picture naming study. Journal of Experimental Psychology: Learning, Memory, and Cognition. Advance online publication. https://doi.org/10.1037/xlm0001049 | Thank you for this valuable comment. You are right, we should discuss our previous study (Döring et al., 2021) in greater detail to 1. make the reader aware that there is another continuous picture naming study that systematically manipulated morphological complexity, and 2. explain why that study cannot answer the question we set out to answer in the current paper.  In our previous study (Döring et al., 2021), we investigate whether compounds’ lemma representations consist of a single lemma (holistic lemma for the compound) or of multiple lemmas (holistic lemma as well as separate lemmas for each constituent) and observed weaker interference for compounds than for simple nouns.  However, the compound targets differed fundamentally to those of the current study, making it impossible to draw inferences from the previous study to the question we set out to answer in this paper.  The categorical relationship of the compounds in the previous study (Döring et al., 2021) was solely established through the **first** constituent, namely their modifiers (**dog** leash, **zebra** crossing, **pony**tail, …), while their second constituent (i.e., their heads) and therefore the compounds themselves were not semantically related. Thus, the observed CSI effect for the compounds was only based on the semantic relationship of the modifier constituents. The simple noun control condition in that study consisted of the compound’s modifiers as free-standing words (**dog, zebra, pony**). We predicted (and observed) a weaker CSI effect for compounds because modifier-related effects are likely to be weaker than head-related or whole-word effects. A compound’s grammatical features are determined by its head, which also carries the bulk of the meaning of a semantically transparent compound (e.g., Zahnbürste [toothbrush]), while the modifier merely provides further specification (i.e., a toothbrush is a type of brush, used for teeth; cf. Bisetto & Scalise, 2005; Zwitserlood, 1994). As a consequence, any effects related to the modifiers of transparent compound targets are likely to be rather weak, which may be why some studies did not observe modifier-related effects at all (e.g., Lorenz et al., 2018a; Lüttmann et al., 2011), while others only found an effect in naming errors (e.g., Lorenz et al., 2018b), or for one very specific type of compound only (e.g., Lorenz and Zwitserlood, 2016).  Thus, the expected and observed difference in CSI between compounds and simple nouns in that study was not due to their different morphological complexity but because the part-word effect in the compound condition was solely induced by the compounds’ modifiers, i.e., the constituent which does not carry the main meaning of the compound. This study, therefore, provides an answer to the question how compounds are represented on the lemma level. It cannot, however, answer the question if the CSI effect differs for semantically related compounds (bookshelf, canopy bed …) and their semantically related simple noun counterparts (shelf, bed …). This is what we tested in the current study. Here, the semantic relationship of the compounds was established through the **second** constituent, i.e., their heads (book**shelf**, canopy **bed**, arm**chair,** …), meaning that the compounds themselves were semantically related. Comparing these to the simple noun control condition consisting of the compounds’ heads gives us the opportunity to find out whether and how the two word types differ in terms of the CSI effect.  Thus, the results of the two studies complement rather than contradict each other.  We now included a brief description of the study in the introduction and slightly reordered the chapter to make the purpose of this study clearer.  We hope this will eliminate any questions and/or reservations you had. |
| Aren’t compound words much lower in lexical frequency than simple nouns? For example, according to the SUBTLEXus norms, the frequencies for ‘shelf’ and ‘bookshelf’ are 6.96 vs. 0.33, respectively. Starreveld et al. (2013) reported an interaction between lexical frequency and semantic relatedness in the picture-word interference task, with a smaller interference effect for high frequency distractors. Might not a similar interaction occur in continuous naming, so that CSI for simple nouns would be expected to be smaller? One way of addressing this would be to include lexical frequencies for both word types in the analyses.  Starreveld, P. A., La Heij, W., & Verdonschot, R. (2013). Time course analysis of the effects of distractor frequency and categorical relatedness in picture naming: An evaluation of the response exclusion account. Language and Cognitive Processes, 28(5), 633–654. | Yes, correct. German compounds are usually much less frequent than their simple noun counterparts.  To account for frequency related effects on naming latencies (Exp1), we initially included lemma frequency (extracted from the dlexdb.de database) into the analyses, as mentioned in the manuscript (p.15, l.370):  “*The fixed structure of the initial model also included the covariates Lemma frequency and Word length. Neither of them improved model fit and were thus excluded from the analysis.”*  With regards to your second question: We believe that frequency does not affect the CSI effect found in continuous picture naming as it might affect interference in the PWI paradigm. While the distractor and target occur in close proximity in PWI, several unrelated items intervene between category members in continuous naming. As Starreveld et al. (2013) report a decreasing frequency effect with decreasing SOA (see Table 1 on page 642), it seems unlikely that this time-sensitive effect survives multiple unrelated items.  So, although we did not expect frequency to influence the CSI or CSF effect, we re-ran the main models of Exp 1 and Exp 2 including an interaction of Ordinal position and frequency to test these hypotheses. The results are as follows:  **Exp1:**  The interaction between Ordinal position and lemma frequency was not significant (t=0.610, p=0.54), meaning that the frequency of the targets did not significantly influence the interference effect found in Exp1.  **Exp 2:**  The results in Exp 2 mirror those of Exp 1, in that lemma frequency does not interact with the facilitation effect (t = -0.53, p=0.59).  We decided not to include these additional analyses into the manuscript as we 1. did not have specific predictions with regards to frequency effects, and 2. do not want to distract from the key findings by adding additional analyses into the analysis and results sections. We hope you agree. |
| On p. 24, the authors state that CSI differed for natural and man-made objects, but this analysis is not reported in the Results section for Experiment 1.  More importantly, Belke (2013) did not report stronger facilitation for natural than man-made objects during superordinate classification. Rather she reported the opposite: participants were faster to classify man-made objects. Riley et al. (2014) also reported that man-made objects were classified more quickly. Hence, the results from Experiment 2 are also not consistent with prior work. In the object recognition literature, faster superordinate classifications observed for man-made objects are typically attributed to them having fewer features than living/natural objects (see Clark & Tyler, 2015). Is it possible that the manmade stimuli employed here actually contain more features and so took longer to process (e.g., does the picture of a bookshelf also show books, making it more complex than just a picture of an empty shelf)?  Clarke, A., & Tyler, L. K. (2015). Understanding what we see: How we derive meaning from vision. Trends in Cognitive Science, 19, 677-687. | We apologise for a mistake that escaped our attention. The statement you are referring to was part of the discussion of Exp 2. By accident, we used the term “interference” when really talking about the observed facilitation. We now correct this mistake.  “*Interestingly, the results also show that natural and man-made items induced different levels of* ***facilitation****, with* marginally *stronger cumulative* ***facilitation*** *for the former than the latter*” (Manuscript, page 26, line 637ff).  Please note: We changed the wording above to “marginally stronger” as we only found a marginal effect (*t* = 1.75, *p* = 0.081) for the interaction Ordinal position * Classification type in our new analysis of Experiment 2 (see Table 2, page 24), compared to the significant interaction we found in the previous analysis we reported in the previous version of the manuscript (*t* = 1.984, *p* = 0.047). Based on the comments we received from Eva Belke (2^nd^ reviewer), we slightly changed the random structure of all our models, nesting the random factor Items under the random factor Category. This resulted in a final random structure that also included by-participant slopes for the factor Classification type, which was not included in the previous version of the model reported in the previous manuscript. However, while accounting for by-participant differences for Classification type only resulted in a marginally significant interaction for Classification type and Ordinal position, it still indicates a possible difference in processing natural and man-made items, which is why we decided to keep the discussion of this effect in the discussion section.  With regards to your second remark, we must disagree. While Belke (2013) did indeed report faster response times for man-made than natural items (main effect), she also reported stronger facilitation for natural than man-made items:  “*Indeed, participants were faster to respond to man-made (…) than to natural entities (…), and the* ***list position effect was less steep for man-made entities than for natural entities*** *(…), yielding a significant interaction of stimulus type (man-made, natural) and list position (…). Apparently, participants tended to respond ‘‘man-made’’ by default,* ***which dampened the linear effect seen in the response times to man-made stimuli relative to that seen for natural stimuli***” (Belke, 2013, p. 236f.)  Thus, our data are consistent with the results reported by Belke. And while we did not find the main effect for Classification type she reports, Riley and colleagues also only observed a main effect in their ANOVA analysis, but not when using a linear-mixed model to analyse the data (i.e., the same analysis we used).  “*We also observed significant effects according to response type in the conventional ANOVA, indicating non-living items were categorized more quickly (∼20 ms) than living items overall, consistent with Belke’s (2013) findings. However, the effect of response type was not significant in the linear mixed effects model, suggesting the findings with the conventional ANOVA approach might be due to averaging of data across items or participants, and so are not reliable*” (Riley et al., 2015, p. 7)  Overall, our data are thus very much in line with previous categorisation studies. |
| The unexpected finding that stronger cumulative facilitation was associated with weaker interference, but only for simple nouns, also requires some more explanation. As the classification task was always run after the naming task, weaker interference predicts stronger facilitation, not the other way round. Participants may simply have benefited from the repeated exposure to the target concepts. Note that Belke (2013) counterbalanced her order of naming and classification tasks. | Yes, we fully agree with you. Our previous explanation of the unexpected finding was not specific enough and did not properly explain the direction of the effect.  We now reran the analysis to investigate the relationship between cumulative facilitation and interference with the order in which the two tasks were completed taken into account. Based on comments from Eva Belke (see below), we now discuss the findings in greater detail (page 33ff). However, while we provide a possible explanation in our manuscript, it is still important to mention that more research is necessary to fully understand the interplay of the two effects. In particular, until there is a computational implementation of the working model proposed by Belke (2013), the explanation remains speculative. We have mentioned this in the manuscript at the end of the section (p.35). |
| **Minor concerns**  One methodological difference also precludes a clearcut interpretation. In the typical continuous naming paradigm, there is no familiarisation phase. Here, the authors employ a familiarisation phase to ensure the correct compound or simple noun was produced. In the picture-word interference paradigm, inclusion of a familiarisation phase has been reported to influence the direction and magnitude of semantic effects (e.g., Collina et al., 2013; Gauvin et al., 2018). What effects on conceptual and/or lemma activation levels might inclusion of this familiarisation phase have produced in the continuous paradigm? Belke (2013) introduced a familiarisation phase but Riley et al. (2014) did not, and both reported identical results for semantic classification, suggesting this factor might not be important for CSI with frequently used, simple nouns.  However, familiarisation might have affected the activation levels of the compounds, if they are less frequently used (see comments above, and Miozzo & Caramazza, 2003 for evidence that familiarising participants with low frequency words reduces the magnitude of the frequency effect).  Collina, S., Tabossi, P., & De Simone, F. (2013). Word production and the picture-word interference paradigm: The role of learning. Journal of Psycholinguistic Research, 42, 461-473. Gauvin, H. S., Jonen, M. K., Choi, J., McMahon, K., & de Zubicaray, G. I. (2018). No lexical competition without priming: Evidence from the picture-word interference paradigm. Quarterly Journal of Experimental Psychology, 7, 2562-2570. Miozzo, M., & Caramazza, A. (2003). When more is less: A counterintuitive effect of distractor frequency in the picture-word interference paradigm. Journal of Experimental Psychology: General, 132(2), 228–252. | In initial continuous picture naming studies, participants were indeed not familiarised with the material (e.g., Howard et al., 2006, Costa et al., 2009). Since then, however, numerous continuous naming studies that did include a familiarisation phase reported identical results to the above-mentioned studies, namely robust cumulative interference (Belke, 2013; Döring et al., 2021; Kuhlen & Abdel Rahman, 2017, 2021; Rose & Abdel Rahman, 2017). Additionally, Kleinman et al. (2015) observed robust cumulative interference in two experiments that differed with respect to familiarisation (Exp 1 and 2) and did not report any differences in strength of cumulative interference between the two. Overall, this suggest that familiarising participants with the material does not significantly influence the cumulative interference effect. We believe our results can thus be interpreted without reservations.  We do, however, agree with your assumption that the familiarisation phase likely heightened the activation levels of the targets in our study. This probably resulted in shorter naming latencies in the first naming cycle (Presentation 1) than would have been observed if we had not familiarised our participants. In fact, this is what Kleinman et al. (2015) report in the above-mentioned study (shorter overall naming latencies after familiarisation (Exp2) compared to no familiarisation (Exp1)).  In our study, this effect was probably larger for low-frequency than for high-frequency words. After all, we found a significant interaction of word type and repetition in Exp1, suggesting that low-frequency compounds benefitted more from repetition priming than high-frequency simple nouns. Thus, the familiarisation may have reduced any frequency effect on overall naming latencies (as you suggested), explaining the frequency-related results we report in our comment above.  As for the classification task: Like you, we believe that the results of previous studies (Belke, 2013; Riley et al., 2015) suggest that familiarising participants with the material does not significantly influence cumulative facilitation. It is likely that familiarisation influences overall response latencies, as in picture naming, but, looking at the results by Belke and Riley et al., it does not seem to influence the effect itself. |
| Did lag or serial position influence any of the results? On p. 12 lag is manipulated by 2, 4, 6, or 8 items but the results section doesn’t mention if lag was included as a covariate in any of the models. Although lags < 8 shouldn’t result in different CSI (see Schnur, 2012), it is still important to check. Similarly, linear effects due to serial order/time on task should be checked per Howard et al. (2006).  Schnur, T. T. (2014). The persistence of cumulative semantic interference during naming. Journal of Memory and Language, 75, 27–44. | Trial number (i.e., serial order) was included as a covariate in all linear-mixed models to account for trial-by-trial sequential effects (i.e., general slowing / learning effects during the experiments). While we mentioned this in the methods section of the previous version of the manuscript, we did not explain the reasons for including trial number as a covariate, which made it easy to miss. We rectified this in the current version.  “*Picture fit (for each item: mean values from picture/label-fit rating) and Trial* ***(consecutive trial number)*** *were included as covariates.* ***The latter was included to account for changes in the course of the experiment (i.e., trial-by-trial sequential effects,*** e.g., 53,56,63***).”*** *(Manuscript, page 15, line 377ff)*  With regards to lag: As previous studies showed that CSI is not influenced by lags of 2-8 (e.g., Belke, 2013; Howard et al., 2006; Schnur, 2014), we did not investigate lag before and did therefore not report it in the previous version of the manuscript.  However, upon your suggestion, we now ran additional analyses for both experiments, adding Lag as a predictor to investigate its influence on interference and facilitation.  We decided to include these analyses in the current version of the manuscript as they provide additional insights on both effects, cumulative interference and cumulative facilitation, and thus are of value to the scientific community. Thank you for pointing out these missing analyses. Including them truly adds to the value of the current manuscript.  Lag in picture naming:  Analysis: page 15  Results: page 16f  Discussion: page 19  Lag in classification task:  Analysis: page 21  Results: page 24  Discussion: page 27 |

Reviewer 2#: Eva Belke

The authors report the results from a three-part continuous naming and classification experiment requiring participants to first name a continuous item list four times, then classify it four times and finally name it once again. Participants named half of the items with more specific compound names and the other half with simple nouns, which they had been familiarized with prior to the experiment. There were consistent cumulative semantic interference effects in each of the first four naming rounds and the follow-up naming round. They were identical in strength for both simple nouns and compound nouns. The authors take these findings to support the notion that the context effect originates at the conceptual level and comes to effect at the lemma level. An increase in the semantic similarity of the category exemplars led to an increase the semantic interference effect in naming in all four rounds. In semantic categorization, it led to an increase in classification in the first presentation but none of the repetitions, which did not yield any context effect at all.
In a combined analysis of the first two sections of the experiment, the authors used the magnitude of the facilitation effect per category and repetition as a predictor in modelling the reaction times in the naming task. They found that stronger facilitation predicted weaker interference, which seems to run counter to the predictions I have made in Belke (2013). The authors discuss this finding in light of differences between the two tasks and argue that it might be compatible with the notion that facilitation and interference cancel each other out, as predicted by the swinging lexical network account.
As the authors note, I have discussed their data with them before, and reading the paper has been a good opportunity to think about the data again based on all the details of the experimental design. The paper is written well and the data should be published in my view. However, I have a few issues with the data analyses and the discussion of the data in the paper that need to be addressed in my view. I would think that all of them can be addressed in a minor revision.

| **Reviewer’s comments** | **Authors’ responses** |
| --- | --- |
| **1. The correlation of facilitation and interference.** In the supplementary analyses, the authors aim at predicting the context effect in naming from the context effect in classification and find that weaker classification effects yield stronger interference effects. At first sight, this seems to be incompatible with the account put forward in Belke (2013) and, as I will argue shortly, with the Swinging Lexical Network (SLN) account, too. However, there are two points to keep in mind when reasoning about the data:  First, the classification task has different task requirements to those of the naming task, as the authors note in the General Discussion. Accordingly, the kind of facilitation arising in semantic classification is different from that arising in naming, which we cannot access directly. Second, reading up on the details of the design I realized that all participants first named the items and then classified them. If we assume that the context effect in naming and classification is mediated by some form of conceptual learning at the interface of lexical concepts and conceptual features, the information that is learnt is bound to differ between naming and classification due to the different task affordances. While in naming, unique features of the category exemplars are very relevant to performing the task, the classification task is more reliant on features shared across items of a set. Accordingly, the conceptual facilitation seen in semantic classification is not identical to the conceptual facilitation that arises as part of the naming process, and the order in which the tasks are administered matters (see Experiment 5 in Belke,2013) and is a relevant piece of information in accounting for the present data. Indeed, when comparing the non-deviant trials shared between Experiments 1 and 5 in Belke (2013), I found that the semantic facilitation effect was attenuated when the third or fourth item in a set was named (Experiment 5) rather than classified (Experiment 1). Arguably, this was due to the fact that naming required a focus on the non-shared information, which attenuated the facilitation effect on classification.  Applying this reasoning to the present data, it is quite plausible that strong interference effects in naming yield weak facilitation effects in classification and vice versa. Naming items from closely related sets requires speakers to focus on the unique features of a concept, and the retuning of the relevant links arising from this is likely to render the representations of same-category members functionally less similar to each other. Accordingly, the semantic facilitation effect will be less strong than for items that have not been rendered more dissimilar before (by virtue of sharing fewer common features in the first place).  One implication of this is that in the present design, the causal relation between the effects seen in naming and classification actually works in the reverse direction than that implemented in the analyses, that is, if anything, the naming data predict the classification data rather than vice versa.  Using the classification times as predictor of the naming times might therefore not be appropriate. I was wondering whether a plain correlation between the predicted slopes of the effect in naming and categorization would be a more appropriate way of analyzing the data. If the authors used slopes generated from the raw data, they may want to run a partial correlation that takes into account effects of semantic similarity. | Thanks a lot for this valuable feedback and the incredibly detailed explanation of the predictions of the working model.  Your explanation expands on the one we provided in the previous manuscript, where we also rendered the unique vs. shared features responsible for the observed findings. However, our previous explanation did not specifically explain the direction of the results but rather vaguely argued why our predictions did not pan out. This was also criticised by the other reviewer. Thus, taking the order of the two tasks into account provides a reasonable explanation for the direction of the prediction and also helps to understand why semantic similarity influences the facilitation effect in the opposite way to the interference effect (see our comment on semantic similarity below). We included your explanation in the current version of the manuscript (p.33f).  However, when considering the explanation, we noticed that it might not be entirely compatible with the finding that cumulative interference is usually not influenced by repetition. If category members are rendered less similar after naming, should this not result in weaker co-activation between category members after the first naming cycle? Repeating the items should, in turn, be associated with less cumulative interference, if we understood the mechanism correctly. This is, however, not what we observed in the main analysis.  We included this limitation in the current version of the manuscript to make the reader aware that there might still be some loose ends (p. 34f.).  Anyway, we took your and the first reviewer’s advice on the importance of task order when analysing the relationship of the two effects and reran the analysis. We now use the interference effect found in Exp 1 to predict the facilitation effect found in Exp 2.  However, instead of including the interference effect as an additional dependent variable into the analysis of Exp 2 to predict the classification RTs, we ran a linear-mixed model that contains the facilitation effect (in ms) as the dependent variable and the interference effect (in ms) as an independent variable. This way, we directly looked at the relationship between the two context effects, which is more true to the research question we set out to answer (i.e., whether one effect can predict the other). As you will see in the results section (p.25), the direction of the effect is identical to our analysis in the previous manuscript, despite exchanging the independent and dependent variables, i.e., strong interference predicting weak facilitation.  We also ran the correlation analysis you suggested but decided to focus on the linear-mixed model in our manuscript, as it gave us the chance to discuss influencing factors, such as semantic similarity, word type and repetition. Nonetheless, we briefly report the correlation (p.28), whose results is in line with those from the linear-mixed model.  We hope that you now find the analyses more suitable. |
| Either way, I would strongly suggest that the authors work with the context effect/the slope of the first presentation in the classification task only. It is clear that after that, all that needs to be learnt to perform that task has been learnt and RTs reach floor, so the context effects/the slopes for the repetitions are even less valid predictors of the conceptual facilitation seen in naming. | We do understand your reasoning but opted to include all repetitions into the analysis, nonetheless. We did this for two reasons:  1. As the new analyses showed significant facilitation effects also after the first classification cylces (i.e., classification cycles 3-5), we wanted to include all classification cycles to fully understand the impact of repetition on the prediction.  2. Using the first classification cycles only would mean a great loss in power. By using a linear-mixed model for the analysis we were able to account for changes due to repetitions, while still using all experimental data.  Yet, out of interest, we ran the same model only for the first classification cycles (first presentation). While the interaction of Interference effect and Semantic similarity was significant (t = -2.5, p = 0.01), the main effect of Interference effect (t=1.5, 0.14) and none of the other effects were. We do not believe that this is because Interference cannot predict facilitation in the first classification cycles but rather due to a lack of power. In this analysis, only 36 (subjects) * 18 (categories) * 1 (presentation) = 648 datapoints were included. |
| Let me conclude this point by pointing out that, despite the evidence in favor of the reasoning outlined above, it remains somewhat speculative until we have an implementation of a working model at our disposal that can simulate conceptual learning in naming or classification tasks. | Yes, we agree.  This is true for both the working model you proposed in Belke (2013) as well as the SLN proposed by Abdel Rahman and Melinger (2009,2019).  We also mentioned this in the manuscript to make readers aware that a computational implementation is necessary to verify these speculative claims (p. 37, line 1179). |
| 2. Conceptual accumulation account vs. (?) SLN? Related to the previous point, I wonder whether the predictions from the account I have put forward in Belke (2013) and those from the SLN, augmented by a learning mechanism, would be different in any way. After all, the accounts are both based on WEAVER++ as a reference model and both accounts predict that there is conceptual facilitation and lexical interference in naming, with lexical interference overriding conceptual facilitation in the continuous naming paradigm. According to the conceptual accumulation account, the an increase in conceptual facilitation causes a corresponding increase in lexical interference, accounting for why, in net reaction times, there is a continuous linear increase with each item that has been named. The SLN, once augmented by a learning mechanism, would make the same predictions, in my reading. | Yes, this is correct. If the SLN was augmented with a learning mechanism at the conceptual level to also accommodate the cumulative facilitation of the classification task, then we would make the same predictions as the conceptual accumulation account.  However, as the SLN currently stands (i.e., without the learning mechanism), cumulative interference in picture naming is explained slightly differently. The SLN assumes that conceptual facilitation is short-lived and thus is constant in each ordinal position, while the interference builds up cumulatively with each named member.  However, after making lots of changes and additions to the General discussion, we decided to exclude the short section on the SLN from the manuscript. As we did not discuss the SLN in the introduction, adding it to the discussion lengthens this section and adds unnecessary complexity to the already quite lengthy discussion. Also, the SLN was solely designed to accommodate speech production data and thus provides no explicit explanation for cumulative facilitation found in the semantic classification task. Thus, any predictions would be highly speculative. |
| 3. Semantic similarity effects in classification. In the analysis of the data from Experiment 2, semantic similarity is included in a two-way and a three-way interaction but not as a main effect. From all I know about modelling interaction effects, the corresponding main effects should always be included as well, unless there is very good reason to exclude them. I cannot see such reasons here, especially since the main effect was included in the model of the data of Experiment 1.  Once the main effect is included, it would be interesting to see how this impacts on the interaction of ordinal position and semantic similarity.  In the model reported in the paper, this interaction does not reach significance (t < 2 and p = .089) but there is a trend that category exemplars that are more similar to their category neighbors yield LESS facilitation rather than more, which would be compatible with the rationale I have laid out in 1. The semantic similarity : ordinal position interaction further interacts with repetition, and the authors argue that the ordinal position effect was stronger initially for categories with very similar exemplars as compared to categories with less similar exemplars. This does not seem compatible with the trend seen for the two-way interaction; if the authors were right, we should see a *negative* trend in the two-way interaction (ordinal position : semantic similarity) rather than a positive one, if I am not mistaken.  Could the authors maybe plot the three-way-interaction?  My impression from reading the model was that the two-way interaction (less facilitation for more similar category exemplars) vanishes after the first repetition and that this led to the three-way-interaction. | Yes, you are right. Our interpretation of the three-way interaction was incorrect and we apologize for this quite severe mistake.  Semantic similarity indeed influences cumulative facilitation differently to cumulative interference, in that weakly related items induce larger facilitation than strongly related ones (as shown in the visualisation). This effect was marginally significant (t=1.92, p=0.06).    However, the visualisation of the significant three-way interaction of Ordinal position* Presentation * Semantic similarity shows that the influence of semantic similarity grows with each repetition. In fact, when analysing the data separately for each presentation, we only found a significant influence of semantic similarity in the last two presentations (i.e., presentation 4 and 5).  We do want to point out, though, that the overall semantic similarity of our items was pretty high, as we chose our items accordingly (see material section in the manuscript). Thus, we did not systematically manipulate this variable, which is why we prefer not to make semantic similarity the main focus in our manuscript.     |
| 4. Dissipation of the semantic facilitation effect in Experiment 2. I have argued above that the facilitation effect in classification dissipates because the information that is learnt in the first round of classification renders the task so easy that the facilitation effect is not discernable any more, or non-existent. I was wondering whether in addition to that, participants may have become aware of the shared category membership of the items in the lists over time. It did not become entirely clear to me what the order of the familiarization and naming tasks was like, given that the lists were tested blocked by word type.  Is it correct that in Experiment 1, participants first received the familiarization for the first set of 60 items, then named it four times, then received the second familiarization and subsequently named the second set of items four times? In that case, it would probably be quite likely that participants became aware of the subsets included in the lists. Schnur, Roelofs and others have discussed that the design as used by the authors (including a lag-2 condition) may raise participants' awareness of the item sets, and this is even more likely to be the case when the list included only relatively few items. Awareness of the sets is probably of little use for the naming task (as participants still have to individuate conceptually each concept from its category coordinates, see Roelofs, 2018), but it is likely that it impacted on the categorization task. Hence, the fact that the context effect re-appeared in original strength in the naming phase after the categorization task (see p. 24) does not refute this reading of the data, as this phase employed a fundamentally different task for which the superordinate category information alone was not sufficient (see also point 1.). | Yes, your understanding of the procedure is correct and we agree with your comment.  We included this additional explanation into our manuscript when discussing the influence of repetition on cumulative facilitation (page 32). |
| 5. Non-interaction of word type and ordinal position. The authors find no interaction of word type and ordinal position once the effect of semantic similarity is accounted for. I share the authors' view that this provides evidence for a conceptual origin of the context effect. However, one might argue that the intensive familiarization may have turned a genuine naming task into some form of cued recall task that might dampen constituent effects on the selection of the compound name at the lemma level.  Could that be the case, or do compound production experiments always include a familiarization comparable to that used in the present experiment? If so, does this also hold for experiments that provide evidence for lexical competition between the compound and its constituents? | In our last paper (Döring et al., 2021, now discussed in the introduction of our manuscript), we familiarised participants in the exact same way, yet found effects that can only be linked to the activation of the separate constituent lemmas.  Thus, we believe that the processing of the compound targets was not significantly influenced by the familiarisation, apart from general repetition priming, which of course, influenced the low-frequency compounds to a greater degree than the high-frequency simple nouns (see comments to the other reviewer above).  To our knowledge, there are no studies that provide evidence for competition between the compound lemma and the constituent lemmas. |
| 6. In all models reported in the paper, categories and items are fitted as separate random effects even though they are probably highly correlated. Why did the authors not code the random effects as a nested structure (i. e. 1 + word type \| category/item)? This seems more true to the design to me. | Yes, you are absolutely right. Using a nested structure is more true to our design and we have now implemented it in all our models. Thank you for pointing that out. |
| 7. Further to this point, my impression is that some of the predictors are correlated to some extent. Most notably, in the models reported in Tables S2 and S3, the slope of the facilitation effect (i. e., the difference between the RTs at ordinal position 5 and 1) and semantic similarity are included as separate factors, but the facilitation slope is most likely influenced by semantic similarity, as is the interaction between the two factors. It may be useful to assess to what extent such collinearity impacts on the models, not only for models S2 and S3 but all models. | Thank you for this valuable comment.  As we understand it, collinearity of two or more variables (above 0.8) reduces statistical power and increases Type II errors. However, centering continuous variables (which we did) considerably helps with collinearity, particularly between main effects and their interactions with other variables.  Nonetheless, we now checked all models for collinearity and multicollinearity but did not find a high correlation between any predictors (main or interaction term) in any of our models. This was confirmed by the Variance Inflation Factors (VIF), which we used to test for multicollinearity. All predictors showed very low correlations (i.e., all VIFs < 1.6).  Here a more detailed explanation for the two main models of each experiment:  Exp1:  All correlations were < 0.21, with the exception of two: Repetition and Trial showed a moderate correlation of -0.61, and the interactions Word type * Ordinal position and Ordinal position * Semantic similarity are moderately correlated at 3.23.  Exp2:  All correlations were < 0.2, with the exception of two:  Repetition and Trial showed a moderate correlation of -0.39 and the interaction terms Word type * Ordinal position and Ordinal position * Semantic similarity had a low correlation of 0.28. |
| Minor points. The data have not yet been made available but the authors have announced that they will be made available in an OSF repository. | The data together with all scripts can now be found at  https://osf.io/qzynk/ |
| On p. 4, the authors may want to make the distinction between the terms origin and locus more explicit. It is there but may be easily missed by those who have not read about it before. | Thank you. We slightly changed the description in the current version of the manuscript, hoping to have made it clearer now.  “*All existing models agree that the locus of cumulative interference, that is, the level at which it comes into effect and behavioural consequences arise, is the lexical level [3,9,22,28–30]. Here, CSI is interpreted in terms of increasing difficulty to select a target’s lexical representation – from now on called “lemma” [6] - amongst a group of co-activated lemma representations. However, activation within the lexical system is short-lived (e.g., PWI paradigm; [12]) and thus not suitable to explain the longevity and accumulating nature of CSI. Therefore, different learning mechanism have been proposed, where structural changes to the system are responsible for the longevity of the effect.* ***The level at which these occur, meaning the level at which cumulative interference actually has its origin (as opposed to its locus, mentioned above), is still a matter of debate****.”* (p. 4, l. 95ff). |
| On p. 9, I am not sure I can follow the argumentation about the effect co-activated first constituents might have. Does the conceptual co-activation of the unrelated first constituent really reduce the activation in the network pertaining to the concept of the target word?  Is there evidence that suggests this might be the case?  Would the activation in the cohort not be primary, i. e. direct, and the activation in the first constituents indirect, mediated via the lemma level? | If one assumes a complex lemma-structure like the one we discuss in Döring et al. (2021, p.5; part of it depicted below), then one could assume that some of the activation the first constituent concept receives (either directly via the conceptual level or indirectly via the lemma level) dissipates.  Let’s take the example in the figure below: The constituent concept Maus (mouse) would co-activate semantically-related concepts, such as Katze (cat) or Hund (dog). These, however, are not related to the compound and its category members, meaning that they do not add to the activation level of the compounds’ semantic category.    If the target was a simple noun, i.e., Maus (mouse), any co-activation the concepts Hund (dog) and Katze (cat) would directly feedback and add to the activation of the other animals.  We hope we were able to clarify this point. |
| p. 30, hindside -> hindsight | Thank you. We have now corrected this mistake. |
