# Supplementary material for: Cumulative semantic interference is blind to morphological complexity and originates at the conceptual level
Source: PLoS One. 2022 Jun 9;17(6):e0268915. doi: 10.1371/journal.pone.0268915 (PMC9182628; doi:10.1371/journal.pone.0268915)
Supplement: Attachment — Submitted filename: Response_to_reviewers_final_submission.docx [file pone.0268915.s004.docx]

**Response to the reviewers**

| **Reviewer’s comments** | **Authors‘ responses** |
| --- | --- |
| **Reviewer #1:** The authors have address all of my concerns. The novel findings from the additional analyses will make an interesting contribution to the literature. | Fantastic. Thank you. |
| Reviewer #2: I am happy with the revisions, except for one point that I would ask the authors to revise. The authors have picked up and addressed my ideas about how the order of tasks may have affected the results, which I appreciate. On p. 34, they note that:  "While this outlined mechanism accounts for the bulk of our data, it might not be entirely compatible with the finding that cumulative interference robustly survives multiple repetitions. If category members are rendered less similar after naming, this should result in weaker co-activation between category members after the first naming cycle. Repeating the items should, in turn, be associated with less cumulative interference. This is, however, not what we observed [see also 27,28,34]."  This is incorrect, and I can see in hindsight that the way I phrased my ideas in my original review may have been too sloppy and, as a result, misleading:  I have not argued that links to shared features are being weakened after naming; after all, they are typically central to the concept and hence needed for it to be activated and named, even if they are not distinctive. Instead, I have argued that less central, individualizing features are being strengthened after naming, as speakers have to focus on these features when trying to resolve the competition between the target and its competitors.  A target concept that has been named before will be co-activated on the next trial, as will be its individualizing features, rendering it harder for the speaker to focus on the individualizing features of the current target. The more targets of a category have been named, the more such individualizing features will be co-activated, causing the previous targets to be strong competitors and rendering target naming increasingly hard. If this was not predicted by the account, it would not be able to account for the cumulative context effect in the first place.  Strengthening individualizing features of a target also causes repetition priming on the next run through the experiment, so the account can explain both effects. I have not found any interaction of the context effect with repetition/load either in Belke (2013, Experiment 1; see FN 3) and I do not think that this finding is incompatible with the account I put forward in the review.  Unlike naming, classification requires that speakers focus on the shared features, which causes the concept-feature links to these features to be strengthened and leads to cumulative facilitation as well as repetition priming.  I would be grateful if the authors could clarify this point in their final version of the paper. Labeling the strengthening of individualizing features as targets becoming less similar is misleading, and it would be good if the authors revised it accordingly. | Thank you. You are correct. We misinterpreted parts of your explanation, which led to an incorrect conclusion about general repetition effects.  In the current version of the manuscript, we deleted the small conclusion section on repetition effects and changed the main text to make the processes behind the results easier to understand. Now, it should be quite clear and not lead to any misreading.  Manuscript, page 34:  “*In the naming task, focusing on the unique features of a target (i.e., those it does not share with other category members) is key to complete the task, namely to select one specific target from among a group of (co)activated items. After successfully naming the target, the links between its features and its lexical representation would be strengthened (i.e., learning mechanism). In picture naming, this mechanism leads to the observed interference, as the strengthened links render the target a strong competitor in the naming process of a to-be-named category member. In the following classification task, however, the strengthened unique links will make it increasingly difficult to focus on the shared features of the target, which are key to complete the classification task. Thus, categories that induced strong interference would then induce weaker facilitation than if the target had not previously been named (and no unique features had previously been strengthened). This would also explain why we observed weaker cumulative facilitation for more strongly related category members (i.e., high semantic similarity) in Experiment 2.”* |
